# Anesthetic Management of an Infant Presenting with an Intrapericardial Tumor: A Case Report

**Published:** 2018-04

**Authors:** Mitra Golmohammadi, Shahyad Salehi, Rozita Haghi, Mohammad Radvar

**Affiliations:** 1 *Department of Cardiac Anesthesiology, Urmia University of Medical Sciences, Urmia, Iran.*; 2 *Department of Cardiac Surgery, Urmia University of Medical Sciences, Urmia, Iran.*; 3 *Department of Pediatrics, Urmia University of Medical Sciences, Urmia, Iran.*

**Keywords:** *Heart neoplasms*, *Teratoma*, *Pericardial effusion*, *Anesthesia*

## Abstract

Intrapericardial teratomas are rare primary cardiac tumors. These tumors, albeit benign in essence, can be fatal if they exert pressure on the cardiovascular and/or respiratory system. We describe a 34-day-old infant, who needed emergent surgery due to cardiovascular structure compromise. Proper anesthetic and surgical techniques conferred an uneventful postoperative course. Histologic examination confirmed the tumor as an intrapericardial teratoma. At 8 months’ postoperative follow-up, the child had a good developmental status and a normal echocardiogram.

## Introduction

Primary tumors of the heart in infants and children are rare, with an incidence rate of 0.0017% to 0.28%.^1^^, ^^2^ Intrapericardial teratomas are the fourth major subgroup of such tumors.^3^ Cardiac teratomas were first reported in 1890.^4^ Autopsy studies in children have revealed an incidence rate of 0.03% to 0.08%. The ability to detect cardiac tumors noninvasively has led to a clear increase in the diagnosis.^5^^, ^^6^ With an embryonic origin, teratomas are composed of elements derived from the 3 germinal layers in varying degrees. If at least 50% of the tumor is comprised of well-differentiated elements, the tumor is referred to as a mature teratoma.^3^ These tumors are usually benign, and their clinical signs and symptoms depend on the tumor size and location.^7^ They may be diagnosed during fetal life (often in the anterior mediastinum), and they are usually associated with pericardial effusion and hydrops fetalis, or soon after birth with severe cardiorespiratory system compromise.^6^^, ^^8^

## Case Report

**Figure 1:**
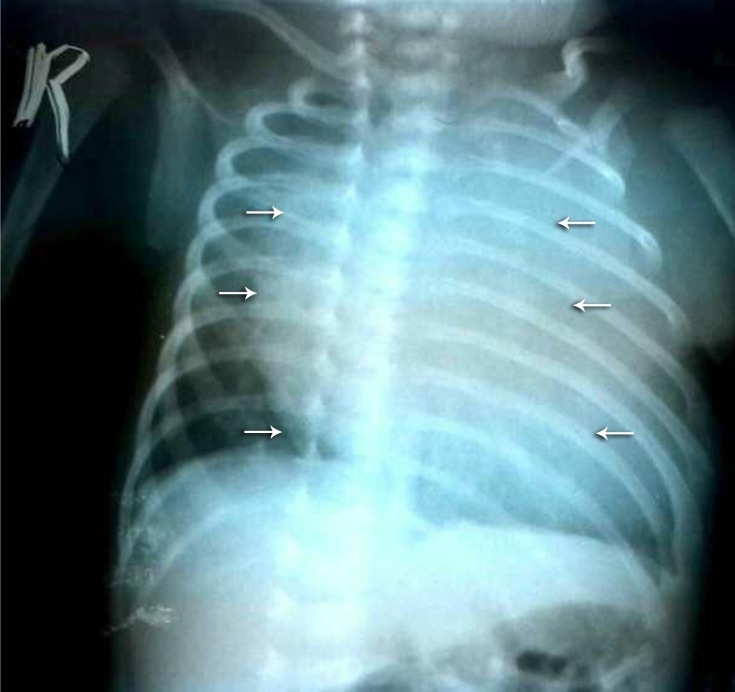
Chest radiograph )anteroposterior view), revealing cardiomegaly and evidence of a pericardial mass. The arrows show the border of the possible mass.

A 34-day-old female infant, weighing 2.3 kg, was admitted to our institute with severe respiratory distress. Physical examination demonstrated severe tachypnea (respiratory rate = 35-40/min), sweating during breastfeeding, peripheral cyanosis, and indrawing of the chest cavity with inspiration. Chest X-ray revealed only cardiomegaly (Figure 1). Transthoracic echocardiography showed massive pericardial effusion with a space-occupying mass lesion, 5 × 4 cm in size, in the pericardial cavity on the right side. Based on the infant’s clinical condition and the echocardiographic findings, she was scheduled for emergency tumor removal. 

**Figure 2:**
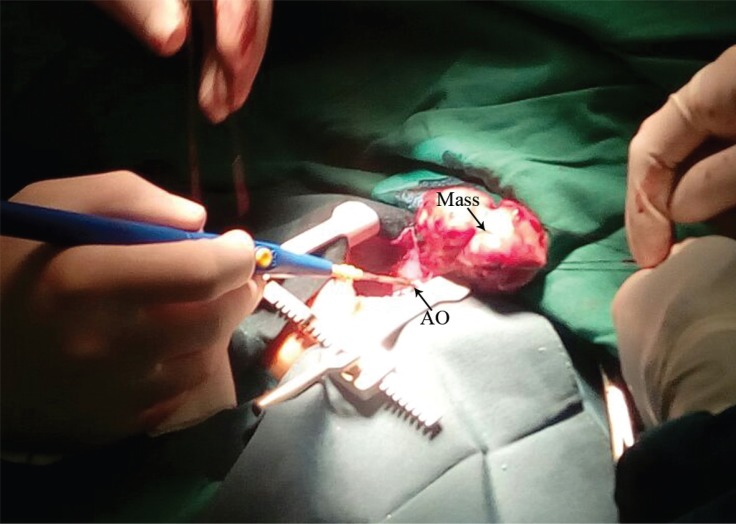
Intraoperative image of the teratoma after opening the pericardium, showing that the mass is adherent to the AO.

The preoperative heart rate was between 130 and 140 bpm. The peripheral capillary oxygen saturation (SpO_2_) before induction was 88% and after the administration of 100% oxygen with a mask, SpO_2_ rose to 94%. Considering the infant^’^s age, severe respiratory distress, and cardiovascular instability, we performed awake-sedate intubation after the administration of 5 μg of fentanyl and topicalization of the tongue, larynx, and vocal cords with 15 mg of 1.0% lidocaine and a 3.0-mm Portex tracheal tube. Subsequently, the general anesthesia was maintained with a mixture of oxygen/air, isoflurane, 1-mg increments of atracurium, and 5 μg of fentanyl intermittently. Thereafter, an arterial line and a central venous line were established. Other monitoring modalities such as the bispectral index (BIS), capnograph, and pulse oximetry were utilized as well. Extracorporeal circulation was on standby. Via a median sternotomy with the preservation of the thymus gland, the pericardium was opened and a large quantity of cloudy yellow fluid was aspirated. A multicystic lesion, around 50 × 45 × 30 mm in size, was revealed. It covered most of the right ventricle and pressed on the right atrium (Figure 2 and Figure 4). Furthermore, the mass was polycystic and attached to the right anterior wall of the ascending aorta. The whole tumor was successfully removed. The patient was extubated in the intensive care unit on the first postoperative day and was discharged from the hospital on the fifth postoperative day.

AO, Ascending aorta

**Figure 3:**
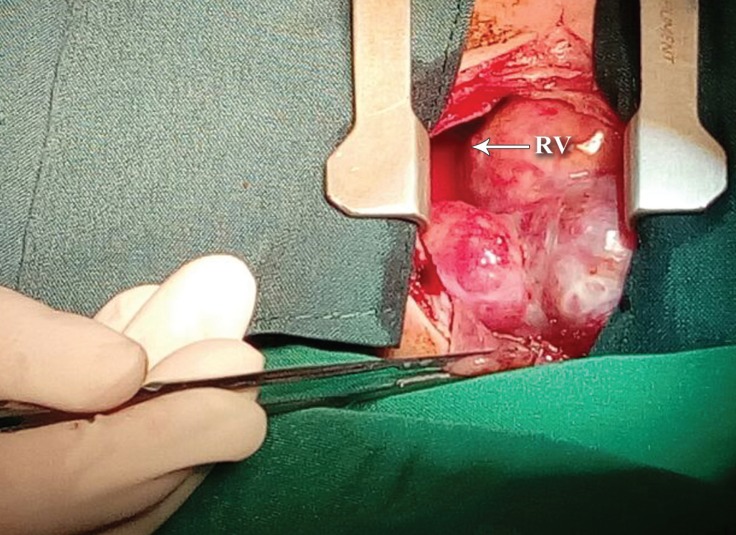
Intraoperative view of the large intrapericardial teratoma, showing that it covers most of the RV.

RV, Right ventricle

**Figure 4:**
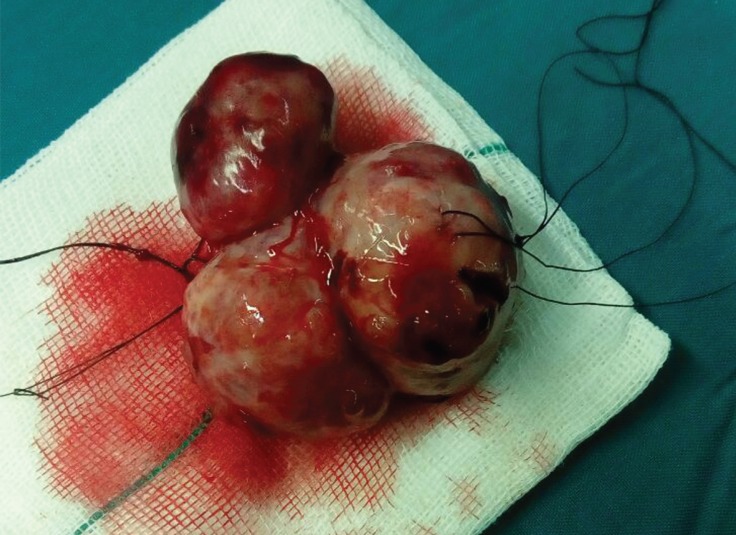
Macroscopic appearance of the mass (multicystic lesion)

Histologically, it was a mature teratoma with the representation of all 3 germinal layers: endoderm 50%, mesoderm 25%, and ectoderm 25%.

## Discussion

Intrapericardial tumors are usually diagnosed in neonates and infants; nonetheless, they may be discovered in the fetal life period. Typically, not only are they lone tumors but also they are large and polycystic with a capsule and a pedicle. Tumor adhesion to the heart itself and/or the great vessels (aorta or pulmonary arteries) is relatively common.^9^^, ^^10^ Most of the time, the tumor is benign and often associated with massive pericardial effusion, cardiac compression, and even severe cardiorespiratory distress.^11^ Herein we described a patient with a pericardial tumor who developed massive pericardial effusion and severe respiratory distress. The hypothesis is that a small patent communication between the cyst and the pericardium results in pericardial effusion and this connection is later on spontaneously sealed by obliterative fibrosis.^12^ Echocardiography is the first imaging diagnostic tool, although magnetic resonance imaging has a better ability to illustrate the anatomical relationship between the intrapericardial tumor and the adjacent vital structures.^13^ Intrapericardial tumors perpetually pose a challenge to both anesthesiologists and surgeons. Anesthesiologists should expect airway obstruction or hemodynamic instability during the induction of anesthesia, which may be unresponsiveness to any treatment except the immediate excision of the mass. However, hemodynamic instability may occur during heart and tumor manipulation.^14^^, ^^15^ Large pericardial tumors may act as an anterior mediastinal mass, which could press the lung, superior vena cava, and pulmonary artery.^16^ The choice of the anesthetic technique depends on the effects of the tumor on the patient’s hemodynamic and clinical status. There is some evidence lending support to the use of specific anesthetic methods.^17^^, ^^18^ Since our patient was a 34-day-old infant with severe respiratory distress and cardiovascular instability, our anesthetic management was awake-sedate intubation without the use of bag-and-mask-ventilation and loss of consciousness. Subsequently, we maintained the general anesthesia with a mixture of oxygen/air, isoflurane, muscle relaxant, and fentanyl. Fortunately, we encountered no unpleasant event during the perioperative period.

 In our patient, although the teratoma was anchored to the ascending aorta and thus caused pressure on the right atrium, we achieved its complete removal. The postoperative period was uneventful, with the patient being extubated on the first postoperative day in the intensive care unit and then discharged home on the fifth postoperative day. Microscopic examination of the specimen yielded a definitive diagnosis. The surgical removal of such tumors is not only curative but also lifesaving because failure to achieve a prompt removal of these lesions can prove fatal. Our patient’s prognosis was good insofar as at 8 months’ postoperative follow-up, she had a good developmental status and a normal echocardiogram.

## Conclusion

Intrapericardial teratomas are rare; they should, however, be included in the differential diagnosis of a newborn or infant presenting with respiratory distress. Intrapericardial teratomas should be removed as soon as they are detected because, despite their inherent benignity, they can be potentially fatal.

## Notes:

***This paper should be cited as: ***Golmohammadi M, Salehi S, Haghi R, Radvar M. Anesthetic Management of an Infant Presenting with an Intrapericardial Tumor: A Case Report. J Teh Univ Heart Ctr 2018;13(2):76-79.
